# Investigating Acid Production by *Streptococcus mutans* with a Surface-Displayed pH-Sensitive Green Fluorescent Protein

**DOI:** 10.1371/journal.pone.0057182

**Published:** 2013-02-28

**Authors:** Lihong Guo, Wei Hu, Xuesong He, Renate Lux, Jeff McLean, Wenyuan Shi

**Affiliations:** 1 School of Dentistry, University of California Los Angeles, Los Angeles, California, United States of America; 2 State Key Laboratory of Microbial Technology, College of Life Science, Shandong University, Jinan, China; 3 J. Craig Venter Institute, San Diego, California, United States of America; University of Kansas Medical Center, United States of America

## Abstract

Acidogenicity and aciduricity are the main virulence factors of the cavity-causing bacterium *Streptococcus mutans*. Monitoring at the individual cell level the temporal and spatial distribution of acid produced by this important oral pathogen is central for our understanding of these key virulence factors especially when *S. mutans* resides in multi-species microbial communities. In this study, we explored the application of pH-sensitive green fluorescent proteins (pHluorins) to investigate these important features. Ecliptic pHluorin was functionally displayed on the cell surface of *S. mutans* as a fusion protein with SpaP. The resulting strain (O87) was used to monitor temporal and spatial pH changes in the microenvironment of *S. mutans* cells under both planktonic and biofilm conditions. Using strain O87, we revealed a rapid pH drop in the microenviroment of *S. mutans* microcolonies prior to the decrease in the macro-environment pH following sucrose fermentation. Meanwhile, a non-uniform pH distribution was observed within *S. mutans* biofilms, reflecting differences in microbial metabolic activity. Furthermore, strain O87 was successfully used to monitor the *S. mutans* acid production profiles within dual- and multispecies oral biofilms. Based on these findings, the ecliptic pHluorin allows us to investigate *in vivo* and *in situ* acid production and distribution by the cariogenic species *S. mutans*.

## Introduction

*Streptococcus mutans* is generally considered to be the principal causative agent for dental caries (tooth decay) [1], [2]. This bacterium can generate acids from fermentable sugars (acidogenicity), which is the main virulence factor in the etiology of dental caries development [3]. This acidogenicity in combination with its ability to survive in the acidic environment it produces (aciduricity), further provides a competitive edge for *S. mutans* over other commensal species [4]. While these important virulence features have been studied in mono-species experiments [3], [4], little is known about the spatio-temporal aspects of acid production and how it is influenced by the presence of other species in the complex multi-species microbial communities that inhabit the oral cavity.

Several techniques have been developed to analyze pH changes within biofilms, including in-dwelling or micro-touch electrodes, fluorescent dyes, and NMR microscopy. In-dwelling electrodes were used to measure the pH at the base of biofilms developed on teeth [5], but their size limited the ability to accurately detect spatial pH changes in the microenvironment of oral biofilms. Micro-touch electrodes that have been employed for several biological systems [6], [7] exhibited similar limitations due to their tip size [8]. Furthermore, both devices are difficult to position and prone to disturbing the very microenvironment that they are supposed to monitor. More recently, pH-sensitive fluorescent dyes as well as various modern imaging systems including laser-induced fluorescence lifetime imaging, two-photon microscopy and NMR microscopy have been used to overcome some of these problems and several studies have demonstrated their utility in measuring pH profiles in microbial biofilms [9]–[15].

An interesting alternative to above approaches are the pH-sensitive green fluorescent protein variants (pHluorins), developed by Miesenbőck *et al.* [16]. Chimeras consisting of a pHluorin and the proteins of interest have been used as *in vivo* pH sensors in a variety of biological systems such as neurons and yeast [17]–[22] to signal not only the anatomical positions of tagged cells or proteins but also their physiological states. In particular two classes of pHluorin have been engineered, which are termed ‘ratiometric’ and ‘ecliptic’ pHluorins. Ratiometric pHluorin produces two excitation peaks – one that increases in intensity with rising pH and one that decreases with rising pH, whereas ecliptic pHluorin gradually loses fluorescence with decreasing pH [23], [24]. Despite the wide application of ecliptic pHluorin as *in vivo* pH sensor in various eukaryotic systems, it was rarely used in bacteria [25]. In fact, to the best of our knowledge, it has never been used as *in vivo* pH sensor for microbial physiological studies under biofilm conditions.

In this study, we explored the application of ecliptic pHluorin to monitor acid production by *S. mutans* in a biofilm environment. An ecliptic pHluorin was fused to the *S. mutans* SpaP protein for *in vivo* expression on the cell surface and the resulting strain was used to simultaneously monitor the localization of *S. mutans* and track the local extracellular pH in an *in situ*, real time manner.

## Materials and Methods

### Bacterial Strains and Growth Conditions

*E. coli* strain DH5α was used for cloning and plasmid amplification. The bacteria were grown aerobically at 37°C in Luria-Bertani (LB) medium supplemented with 250 µg/mL spectinomycin or 100 µg/mL ampicillin when needed for plasmid selection. *S. mutans*, *S. sanguinis* and *S. gordonii* were routinely cultured in Todd-Hewitt (TH) media (Difco) at 37°C in the presence of 5% CO_2_. The *S. mutans* strain expressing the pHluorin-SpaP fusion was cultured in the same medium supplemented with 800 µg/mL spectinomycin.

### Strain Construction

**Figure 1:**
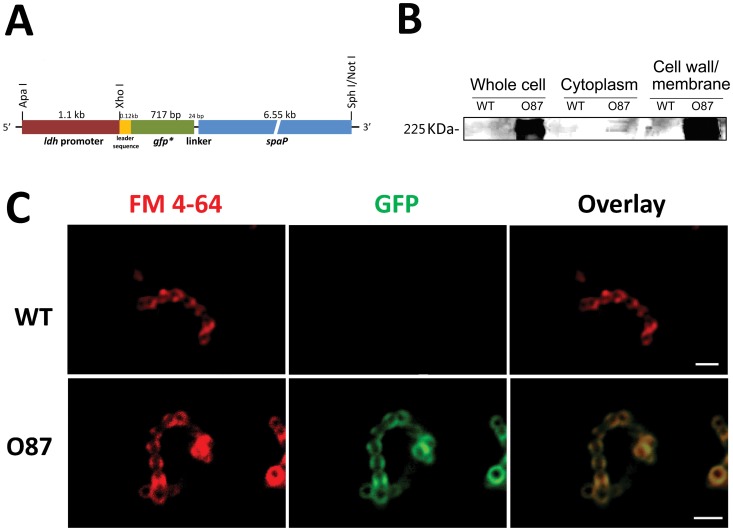
Construction of *S. mutans* O87 with cell surface-expressed pHluorin. (A) *gfp*-fusion construct. The illustration shows the construct for surface-displayed expressed pHluorin in *S. mutans* (strain O87). The ecliptic pHluorin gene was inserted between the second and third amino acids after the identified signal-peptide cleavage site of the surface protein SpaP to enable orientating the pHluorin onto the cell wall of *S. mutans*. (B) Western blot with a GFP antibody shows the presence of the surface-expressed chimeric pHluorin-SpaP fusion protein (225 kDa) in *S. mutans* strain O87. The different cell fractions are indicated above each lane. (C) CLSM analysis of pHluorin localization in *S. mutans* strain O87, and wild-type cells (WT) served as control. Membrane staining with the lipophilic fluorescent dye FM 4–64 is shown in red (*left*), GFP signals are shown in green (*middle*), and the overlay images on the right. The images were taken with a 63×objective and deconvoluted to resolve cellular localization. The scale bars represent 2 µm.

The backbone vector for constructing gene fusions was pBluescript II SK (-), and pFW5 was used as suicide vector for integration of the recombinant *gfp-spaP* fusion into the *S. mutans* genome. To construct a cell surface displayed ecliptic pHluorin derivative, the 0.7 kb ecliptic pHluorin encoding ORF [16] was amplified by PCR from vector pGM87 (kindly provided by Dr. Gero Miesenbőeck) using primers phluorin-01 and phluorin-02 (Table 1). The pHluorin coding sequence with a linker sequence (accggtcccgccgcttccgccgct) at its 3′ end was then in-frame inserted via overlapping PCR between the second and third amino acids after the identified signal-peptide cleavage site of SpaP [26], a surface protein antigen-encoding gene of *S. mutans*. Three initial PCR reactions were performed to generate overlapping gene segments (leading sequence-*gfp*-linker-*spaP)*. Internal primers ovpcr-b, -c, -d and ovpcr-e (Table 1) generated overlapping, complementary 3′ ends on the intermediate segments. Overlapping strands of these intermediate products hybridize at this 3′ region in a subsequent PCR and are extended to generate the full-length chimeric gene product amplified by flanking primers ovpcr-a and ovpcr-f (Table 1). PCR was initiated by a hot-start procedure and reaction mixture containing all other reagents except *Pfu* polymerase (Stratagene) was heated at 94°C for 10 sec, then brought to an 80°C holding temperature prior to addition of 5.0 U *Pfu* polymerase. Then cycling proceeded as follows: 94°C 10 sec, 68°C 4 min for 15 cycles; then 94°C 10 sec, 68°C, 4 min plus 15 sec extension period per cycle for further 15 cycles; followed by a 10 min extension at 72°C. A 1.1 kb DNA sequence upstream of the *ldh* start codon containing the *ldh* promoter [27] was PCR amplified from genomic DNA of *S. mutans* UA 140 using the primers ldh-p01 and ldh-p02 (Table 1). The resulting chimeric gene product was then ligated downstream of the lactate dehydrogenase gene (*ldh*) promoter (Fig. 1A, and a schematic drawing was provided as supporting information).

**Table 1:** Primers used in this study.

Primer	Direction^a^	Sequence^b^	Target gene product	Product size (bp)
ldh-p01	Fw(ApaI)	5′-agggggccctctcgaacagcagtgatacc-3′	*ldh* promoter	1,110
ldh-p02	Rev(XhoI)	5′-cgcctcgagacagcaccatcaccaacaag-3′		
phluorin-01	Fw(XhoI)	5′-cgcctcgagagtaaaggagaagaacttttca-3′	Ecliptic pHluorin gene	723
phluorin-02	Rev(SmaI-SphI)	5′-tcccccgggggacatgcatgctttgtatagttcatccatgcc-3′		
ovpcr-a	Fw(XhoI)	5′-cgcctcgagatgaaagtcaaaaaaacttacgg-3′	Signal peptide and first two amino acids coding gene of *spaP*	120
ovpcr-b	Rev	5′- gaaaagttcttctcctttactttcatcggcaaaaaccttttg-3′		
ovpcr-c	Fw	5′-caaaaggtttttgccgatgaaagtaaaggagaagaacttttc-3′	Ecliptic pHluorin gene and linker	747
ovpcr-d	Rev	5′-tcactagtagtggtcgtaccggtcccgccgcttccgccgctggacatgcatgctttgta-3′		
ovpcr-e	Fw	5′-tacaaagcatgcatgtccagcggcggaagcggcgggaccggtacgaccactactagtga-3′	*spaP* gene absence of signal peptide and first two amino acids coding sequence	4569
ovpcr-f	Rev(NotI-SphI)	5′-ataagaatgcggccgctaaactatacatgcatgctcaatctttcttagcctttaagc-3′		

a Fw, forward; Rev, reverse.

b Restriction site sequences are underlined.

The recombinant construct was confirmed by restriction analysis, PCR, DNA sequencing and testing of the GFP-SpaP reporter protein function. pFW5Φ(*ldh*_p_-leading sequence-*gfp*- linker-*spaP*) were transformed into *S. mutans* strain UA140 to generate pHluorin-expressing strain O87. The integration of the construct into the chromosome of *S. mutans* via single crossover homologous recombination was confirmed by PCR and its functional expression was confirmed by fluorescent microscopy analysis (Nikon ECLIPSE E400 microscope).

Strains, plasmids and primers used in this study are shown in Tables 1 and 2.

**Table 2:** Strains and plasmids used in this study.

Strain	Relevant characteristics	Reference
*E. coli* DH5α	*supE44 lacU169 (80 lacZ* M15*) hsdR17 recA1 endA1 gyrA96 thi-1 relA1 luxS*	[28]
*S. mutans* UA140	Wild-type *S. mutans* Kan^s^ Erm^s^	[29]
*S. mutans* strain O87	UA140: : Φ(*ldh_p_*-leading sequence-*gfp*-linker-*spaP*)	This work
Plasmid		
pGM87	Superecliptic pHluorin gene in pGEX-2T; Amp^r^	kindly provided by Dr. Gero Miesenbőeck
pBluescript II SK (−)	Cloning vector; Amp^r^	[30]
pFW5	shuttle suicide vector; Spec^r^	[31]
pFW5-*ldh_p_*-leading sequence -*gfp*-linker-*spaP*	Ecliptic pHluorin gene under *ldh* promoter, inserted between the second and third amino acids after the identified signal-peptide cleavage site of surface protein antigen SpaP, with a linker downstream of *gfp*; Spec^r^	This work

### Western Blots

Cell wall/membrane preparations from *S. mutans* were processed as described previously [32]. Briefly, cells were grown overnight at 37°C, collected by centrifugation and resuspended in 50 mM Tris•HCl (pH 8.0) containing 1 mM PMSF. After being transferred to a chilled 2 ml microcentrifuge tube containing 425–600 µm diameter glass beads (Sigma), cells were lysed using a Mini-Bead Beater homogenizer (Biospec Products) at full speed for 10 min. The glass beads were removed by decantation and the undisrupted cells were pelleted by centrifugation at 2,000×*g* for 10 min. Half of the supernatant was stored as whole-cell lysate. The remaining supernatant was further centrifuged at 150,000×*g* for 2 h, and the supernatant was collected as cytoplasmic fraction of the cells. The cell wall/membrane fraction containing pellet was washed once with distilled water and resuspended in 50 mM Tris•HCl (pH 8.0) supplemented with 1 mM EDTA and treated with RNase (10 mg L^−l^) and DNase (10 mg L^−l^) at 37°C for 2 h.

Western blotting of whole-cell lysates, cytoplasmic fractions, as well as the cell wall/membrane fractions was performed using anti-GFP antibody (Thermo) [33]. The signal was developed using the Pico chemiluminescence kit (Pierce).

### General Phenotypic Characterization Assays

Growth kinetics were measured for *S. mutans* UA140 and the strain O87 containing the cell surface displayed pHluorin. Biofilm formation, glucan production and glycolytic rate were also performed as previously described [34]–[36].

### Growth of *in vitro* Biofilms

Overnight cultures (OD 600 nm = 1) of *S. mutans* wild type or its derivative were diluted 1∶100 in fresh TH medium containing 0.5% (wt/vol) sucrose. Five hundred µl of diluted culture was inoculated in each well of a sterile 8-well Lab-Tek™ Chambered Coverglass (Nalge Nunc International, Naperville, IL), followed by 16 h static incubation at 37°C in 5% CO_2_ atmosphere to allow mono-species biofilm formation.

To establish dual-species biofilms, 1.25×10^6^ CFU/ml of *S. mutans* was mixed with the base-producing bacteria, *S. sanguinis* ATCC 10556 or *S. gordonii* DL1 [37], [38] at a ratio of 1∶10 and followed by 16 h incubation under same condition as described above for mono-species biofilm formation. The saliva-derived biofilms spiked with *S. mutans* cells were established as follows: Unstimulated saliva samples were collected from 6 subjects 6 h post-cleaning. Individual saliva samples were diluted 1∶4 in TH medium. The suspension was subjected to a low speed centrifugation for 10 min at 600×*g* to remove eukaryotic cells and large debris [39]. Five hundred µl of the supernatant was inoculated into each well of a sterile 8-well Lab-Tek™ Chambered Coverglass, together with about 1.25×10^6^ CFU/ml *S. mutans* cells expressing surface associated ecliptic pHluorin (O87). The ratio of salivary bacteria to *S. mutans* is about 10∶1. Subsequently, 16 h dual-species and saliva-derived biofilms spiked with *S. mutans* were challenged with either 2% sucrose or 50 mM arginine-HCl (pH 7.6).

### Fluorescence Measurement

Fluorescence emission of pHluorin was measured by a Cary Eclipse fluorescence spectrophotometer (Varian, Mulgrave, Victoria, Australia) using a bandpass filter with a center wavelength of 400±5 nm. The fluorescence signal intensity of planktonic cells of *S. mutans* strain O87 with surface-expressed pHluorin was monitored at different pH. The buffer solutions (pH 5.5, 6.0, 6.5, 7.0, 7.5) were prepared by mixing potassium phosphate monobasic anhydrous and sodium phosphate dibasic heptahydrate in different ratios to obtain the desired pH. The pH of the buffer solutions was determined by the AB15 pH meter (Fisher).

### Imaging

All biofilm images were collected with a Zeiss LSM 5 PASCAL confocal laser scanning microscope (CLSM) using LSM 5 PASCAL software (Zeiss, Jena, Germany).

For visualization, the dual-species biofilms and saliva-derived biofilms spiked with *S. mutans* O87 were stained with 5 µM CellTracker Orange CMRA (Invitrogen) according to manufacturer’s instructions. *S. mutans* within biofilms was specifically labeled with an anti-*S. mutans* monoclonal antibody SWLA1–IgG2a as described previously [40]. Alexo Fluor 633 conjugated goat anti-mouse IgG (Sigma, St Louis, MO) was used as secondary antibody.

For evaluating the respiratory activity of microcolonies in *S. mutans* biofilm and semiquantitative estimate of metabolic *vs* dormant cells, 5 mM 5-cyano-2, 3-ditolyl tetrazolium chloride (CTC) (BacLight RedoxSensor CTC Vitality Kit, Invitrogen) [41] was added at the beginning of biofilm formation, and CTC fluorescence was monitored when the biofilms were 16-h old.

Excitation at 488 nm with an argon laser in combination with a 505–530 nm bandpass emission filter was used for pHluorin fluorescence imaging and a 560 nm longpass emission filter for CTC fluorescence imaging. An excitation at 543 nm and 633 nm with helium-neon laser and a 560 nm and 650 nm longpass emission filter was utilized respectively to reveal CellTracker Orange-stained and Alexa Fluor 633-labeled cells within the biofilms. The scanning module of the system was mounted onto an inverted microscope (Axiovert 200 M). The 40×(NA/1.3) or 63×(NA/1.4) numerical aperture oil-immersion objectives were used for imaging. To visualize pHluorin expression with CLSM, cells were left for 1 h at room temperature to aerate the cells for proper folding of the pHluorin fluorophore [42]. The samples were placed on a commercially available thermoelectric heating stage insert (Brook Industries, Lake Villa, IL) (130×95×1 mm) to keep the biofilms at 37°C during imaging. Image stacks of three randomly chosen spots were collected for each biofilm for data analysis.

In order to record pH changes in biofilms following a sucrose spike, an initial series of images was taken as a baseline, followed by addition of sucrose to a final concentration of 2%. The same field was imaged every 15 min over a 1 h time period. Time point t = 0 was measured just before sucrose addition.

### Image Analysis

Image stacks (1024 by 1024-pixel tagged image file format) were quantified using the image analysis software COMSTAT with MATLAB 7.0 (The MathWorks, USA). In order to control for photobleaching, image stacks in a buffered neutral environment were taken. In fact, the decrease of fluorescence intensity of pHluorin due to photobleaching was less than 10% over 15 min. DeconvolutionJ was used to remove or reverse the blurring present in microscopic images and improve the contrast and resolution of digital images.

## Results

### Construction of a *S. mutans* Strain Expressing pHluorin on its Cell Surface

In an effort to display ecliptic pHluorin on the cell surface of *S. mutans* for effective monitoring of local extracellular pH at the individual cell level in a real time, *in situ* manner, we constructed strain O87. In this strain ecliptic pHluorin was fused to the *S. mutans* surface antigen protein SpaP to express a cell wall-associated chimeric protein (Fig. 1A). Western blot analysis with a GFP-specific antibody confirmed that the ecliptic pHluorin was present in the whole cell and cell wall/membrane fractions but not in the cytoplasmic fraction of strain O87 (Fig. 1B). Furthermore, when using the lipophilic fluorescent dye FM 4–64 to reveal the cell membrane, we observed that the pHluorin fluorescence signal of strain O87 colocalized with the membrane fluorescence signal (Fig. 1C).

The integration of pHluorin reporter system into the chromosome of *S. mutans* did not affect cell viability, and no significant difference in biofilm formation, glucan production and glycolytic pH drop was observed between strain O87 and its parent strain (data not shown).

### pH-responsivity of *S. mutans* Cells Carrying the Surface Displayed pHluorin

**Figure 2:**
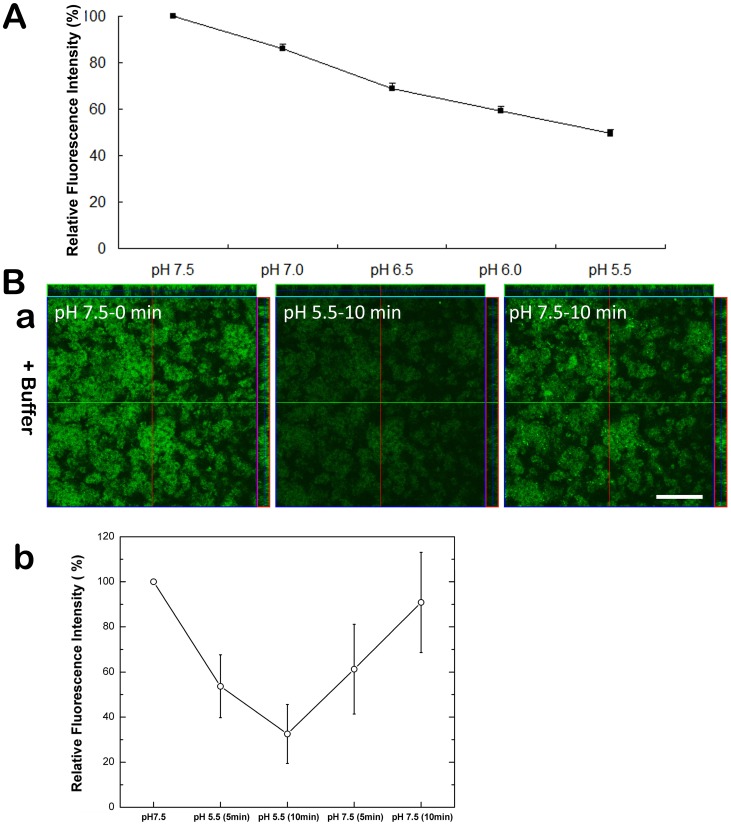
pH-responsivity of surface-expressed pHluorin in *S. mutans*. (A) Fluorescence signal intensity of the pHluorin-SpaP fusion protein in *S. mutans* strain O87 at different pH values. Planktonic cells of strain O87 were adjusted to the indicated pH values. The ratio was calculated as the amount of pHluorin signal at each pH value vs. the initial signal at pH 7.5. The plots show the average of triplicate samples, and the error bars correspond to the standard deviations. (B) Time-course of surface-expressed pHluorin fluorescence signal in *S. mutans* biofilms at different pH values. CLSM analysis of surface-expressed pHluorin signals in *S. mutans* O87 biofilms is shown in (a). The fluorescence of biofilms was monitored at pH 7.5 (left), shifted to pH 5.5 for 10 min (middle) followed by a shift back to pH 7.5 for 10 min (right). Quantification of pHluorin signals in biofilms at different pH is shown in (b). The ratio was calculated as the amount of pHluorin signal at each time point vs. initial amount (0 min). The plots represent the average of three duplicate tests.

To examine the *in vivo* pH-responsivity of the ecliptic pHluorin when expressed on the cell surface of *S. mutans* as a pHluorin-SpaP fusion protein, planktonic cells of strain O87 were suspended in buffer with different pH values and their fluorescence signals were monitored. A reduction in fluorescence signal intensity corresponding to the decrease in pH was observed (Fig. 2A), confirming the pH-responsivity of the surface displayed ecliptic pHlourin in *S. mutans* strain O87 and its potential for detecting pH changes in real time.

The pH-responsivity of the cell surface displayed pHluorin-SpaP fusion protein in strain O87 was further tested under biofilm growth conditions. Similar to their planktonic counterparts, the biofilm cells responded to the shift in external pH from 7.5 to 5.5 with a drastic reduction (∼70%) in pHluorin fluorescence intensity within 10 min. The signal intensity was almost fully restored (>90%) after 10 min exposure to a readjusted external pH of 7.5 (Fig. 2B).

### pHluorin Enables Temporal and Spatial Evaluation of the *in situ* pH Profiles within *S. mutans* Biofilms

**Figure 3:**
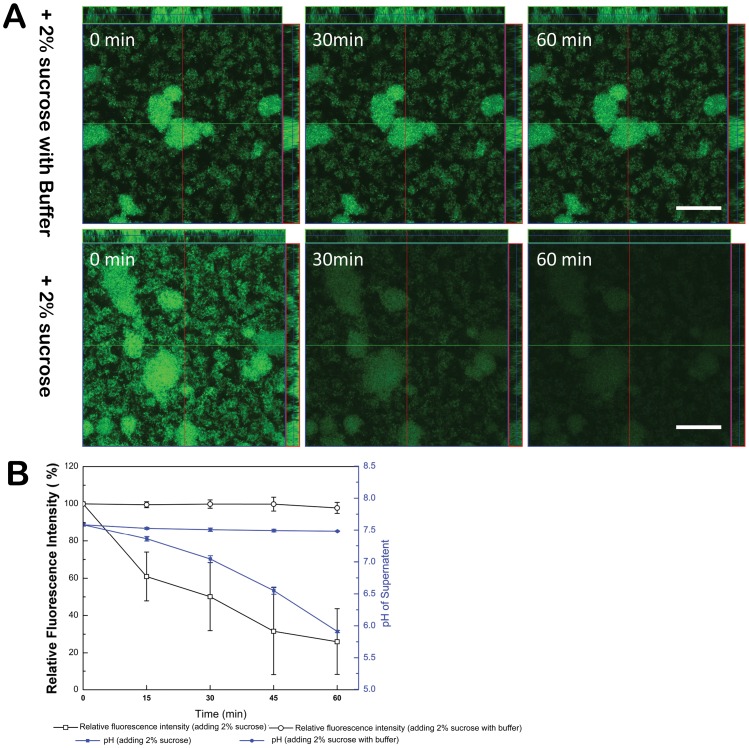
Temporal and spatial distribution of pHluorin fluorescent signals within *S. mutans* biofilms. (A) Dynamic analysis of surface-expressed pHluorin fluorescence signal changes in biofilm after addition of 2% sucrose under phosphate buffered condition (pH 7.5) (upper panel) and unbuffered condition (lower panel), respectively. (B) pHluorin signals after addition of 2% sucrose under buffered or unbuffered conditions is quantified. The proportion was calculated as the amount of pHluorin signal at each time point vs. the initial signal intensity (0 min). The plots show the average of three duplicate tests. The corresponding changes in medium pH are also included.

The dynamic changes of pHluorin-SpaP fusion protein fluorescence intensity within *S. mutans* biofilms were monitored after addition of 2% sucrose under phosphate buffered (pH 7.5) and unbuffered condition (Fig. 3). Results showed that, under buffered condition, the medium pH remained stable at 7.5 and the biofilms displayed bright fluorescence throughout the 60 min incubation period even in the presence of 2% sucrose. In unbuffered medium in contrast, the addition of sucrose induced a pH drop from 7.5 to below 6.0 within 60 min accompanied by a striking decrease in the fluorescent signals within *S. mutans* biofilms. Interestingly, after 30 min of incubation, the pHluorin fluorescence intensity decreased by nearly 50%, although the medium pH only dropped to about 7.0. According to the change in fluorescent intensity observed in response to pH reduction in Figure 2A, the microenvironment pH of microcolonies decreases to almost pH 5.5. This suggests that the pH in the microenvironment decreases prior to the pH drop in the medium following sucrose fermentation.

### pHluorin Disclosing Non-uniform pH Profiles Associated with Differences in Microbial Metabolic Activity

**Figure 4:**
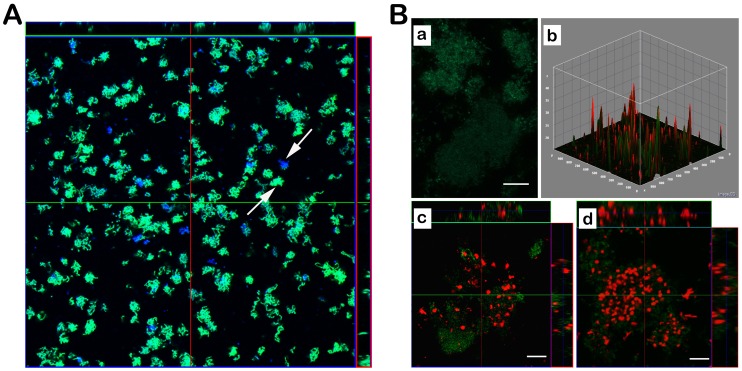
Signals of the pHluorin-SpaP fusion protein and metabolic activities of *S. mutans* in biofilms after addition of sucrose. (A) CLSM image showing pH heterogeneity of different microcolonies (indicated by arrows) within *S. mutans* biofilm in the presence of sucrose. Biofilms were revealed with CellTracker Orange. CellTracker Orange (blue), GFP (green) and the corresponding overly images are shown. (B) CLSM images showing differences in metabolic activities of microcolonies within *S. mutans* biofilm after addition of sucrose. (a) CLSM analysis of pHluorin signals of *S. mutans* strain O87 biofilm after addition of 2% sucrose for 60 min. Bars represent 50 µm. (b) 3D surface plot of fluorescence intensities of pHluorin (green) and the fluorescent metabolic indicator dye 5-Cyano-2,3-ditolyl tetrazolium chloride (CTC - red) in strain O87 biofilm after addition of 2% sucrose for 60 min. (c) and (d) The representative CLSM images of microcolonies within the same biofilm. Bars represent 20 µm.

One intriguing discovery in this study is the pH heterogeneity of different microcolonies within the same *S. mutans* biofilm in the presence of sucrose (Figure 4A). Within biofilms of *S. mutans* O87 some microcolonies displayed a much more intense green fluorescence signal than others (indicated by arrows) after addition of sucrose, suggesting the pH in the microenvironment of microcolonies is not homogeneous throughout *S. mutans* biofilms following sucrose fermentation. To investigate the possible correlation between microenvironment pH heterogeneity and the metabolic status of microcolonies, CTC (5-cyano-2,3-ditolyl tetrazolium chloride) was added to the experimental setup for evaluating the respiratory activity of microcolonies within *S. mutans* biofilm in the presence of sucrose and a correlation between metabolic status and pH was observed (Fig. 4Ba and 4Bb). Microcolonies with less CTC signal typically exhibited brighter pHluorin fluorescence, while strong CTC signals were often associated with reduced pHluorin fluorescence (Fig. 4Bc and 4Bd).

### Surface-displayed pHluorin as a Tool for Monitoring *in situ* Acid Production by *S. mutans* within Oral Biofilms

**Figure 5:**
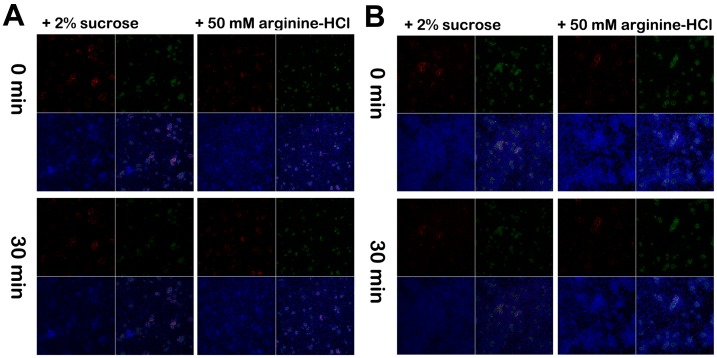
Fluorescence signals of pHluorin-Spap fusion protein **in dual- and multispecies oral biofilms.** Time-course of surface-expressed pHluorin fluorescence signal in 16 h *S. mutans* O87 and *S. sanguinis* dual-species biofilm (A) and saliva-derived biofilms (B) after challenged with 2% sucrose or 50 mM arginine-HCl (pH 7.6). At different time points (0 and 30 min), biofilms were stained with the anti-*S. mutans* monoclonal Ab SWLA1, the secondary Ab Alexo 633 conjugated goat anti-mouse IgG and CellTracker Orange. SWLA1 (red), CellTracker Orange (blue), GFP (green) and the corresponding overlay images are shown in the four small panels.

Next we explored the application of the surface-displayed (O87) pHlourins as “pH-meters” to study acid production by *S. mutans* under biofilm conditions in the presence of other microorganisms. For pHlourin-independent identification of O87 within dual-species biofilms with *S. sanguinis* or *S. gordonii* as well as a multi-species environment, we employed a previously described *S. mutans*-specific monoclonal antibody (MAb) together with a corresponding red-fluorescent labeled secondary antibody. This approach allowed simultaneous monitoring of the spatial localization of *S. mutans* and its acid production *in situ* in dual-species (Fig. 5A) and multi-species (Fig. 5B) biofilms. In the presence of sucrose, acids generated by glycolysis lowered the pH in the microcolonies microenvironment of *S. mutans* as indicated by the corresponding reduction in pHluorin fluorescence intensity. For the dual-species biofilms, the pHluorin fluorescence intensity decreased more significantly after addition of sucrose for 30 min in comparison with addition of arginine (20.0±9.33% *vs* 6.14±12.86% in fluorescence reduction). Similar reduction rates in fluorescence intensity were observed for the saliva-derived multi-species biofilms (16.14±7.16% *vs* 5.52±6.23% in fluorescence reduction).

## Discussion

In this study, we demonstrated, for the first time, the use of a pH-sensitive variant of GFP the ecliptic pHluorin, [16] as an *in vivo, in situ* pH meter to monitor the acid production by cariogenic *S. mutans* at the individual cell level.

SpaP was chosen as a fusion partner to orientate ecliptic pHluorin onto the cell surface of *S. mutans* for sensing extracellular microenviromental pH within biofilms. The surface-anchored chimeric fusion protein displayed similar *in vivo* pH responsiveness characteristics to the original ecliptic pHluorin, indicating that the in-frame fusion of ecliptic pHluorin to SpaP of *S. mutans* retained its spectral properties, and the resulting *S. mutans* O87 strain can be used to monitor extracellular microenviromental pH within biofilms.

Our study demonstrated that, by changing its fluorescence intensity in a pH-dependent manner, *S. mutans* O87 was able to monitor cellular acid production within mono-species biofilm in the presence of fermentable carbohydrates. Intriguingly, when exposed to 2% sucrose for a short period of time, although the solution pH stayed around 7.0, a more drastic reduction in pH (5.5) was monitored within *S mutans* microcolonies indicating the microenvironments within microcolonies experience a much faster pH drop. The exposure of acidogenic bacteria, such as *S. mutans*, to an excess amount of fermentable carbohydrates can result in the rapid accumulation of glycolytic intermediates and acidification of the extracellular microenvironments. However, due to the diffusion barrier of the matrix and buffering capacity of the medium, the pH monitored in the macro-enviroment often does not reflect the acidity within the microbial colonies. For example, in the human oral cavity the pH of saliva collected directly adjacent to plaque remains near neutral, while localized pH within plaque can drop below 5.5 following sugar consumption, and trigger demineralization process [43]. Previous studies also showed that the pH within a biofilm may differ substantially from that encountered in the surrounding solution [11], [20], [44], [45]. Within biofilms, the pH of the local microenvironment is more important and clinically relevant than the overall pH since the intra-plaque pH determines the dental plaque structure and physiological process, and ultimately affects enamel and dentine demineralization [46]. It has been proposed that pH fluctuations in microenvironments could cause a heterogeneous distribution of mineral phases within natural biofilms [47], [48]. Sissons *et al*. pointed out that the pH at any point within dental plaque biofilm resulted from an interaction of cells and cell buffers with bacterial metabolism of a diffusion-limited supply and clearance of substrates, metabolic products and mobile buffers [49]. The presence of extracellular polysaccharides (EPS) within and surrounding microcolonies could restrict the diffusion of a variety of molecules, thus affect the microenvironment pH within the biofilms [12], [15], [50]–[52]. Meanwhile, it is also likely that the *S. mutans* cell surface might retain protons generated from its own acid production to prepare for acid-stress ahead of the rest of the bacteria.

Our study further revealed that pH reduction among *S. mutans* microcolonies was not uniform when biofilms were exposed to sucrose. pH heterogeneity of different microcolonies exists within *S. mutans* biofilm in presence of sucrose (Fig. 4A). While the majority of microcolonies lost their fluorescence due to fermentation-induced pH drop after addition of sucrose for 60 min, there were still microcolonies with fairly strong fluorescence signals (Fig. 4B). The heterogeneity of pH profiles suggested that, within the same mono-species biofilm different cell populations with differential physiological status could coexist. Haagensen *et al*., have shown that the development of *Pseudomonas aeruginosa* biofilms involved an early differentiation of the attached cells into at least two isogenic but clearly distinguishable subpopulations [53]. It has also been demonstrated that *S. mutans* cells could segregate into two subpopulations induced by CSP, one becoming competent and the other lysed, resulting in intrapopulation diversity [54]. The factors that contribute to physiological heterogeneity in biofilms include micro-scale chemical gradients, adaptation to local environmental conditions, stochastic gene expression and genotypic variation that occurs through mutation and selection [55]. Our CTC staining further revealed that the differential acid production of *S. mutans* microcolonies was correlated with their different metabolic activities.

Using the pHlourin expressing strains, we found that pH values on the *S. mutans* surfaces changed when incubated in the presence of oral commensal organisms such as *S. sanguinis* or *S. gordonii*. In the presence of arginine, *S. sanguinis* (Fig. 5A) or *S. gordonii* (data not shown) in the dual species biofilm, or other arginine-utilizing bacteria (Fig. 5B) within saliva-derived biofilm can consequently produce alkali, and the elevated microenvironment pH of microcolonies as a result of alkali production increases the pHluorin fluorescence intensity. While in the presence of sucrose, *S. sanguinis* or *S. gordonii* in the dual species biofilm, or other acidogenic bacteria such as lactobacilli within saliva-derived biofilm also metabolize carbohydrate to acid besides *S. mutans*, and will reduce the microenvironment pH of microcolonies as a result of acid production decreases the pHluorin fluorescence intensity. The results suggest that the surface-anchored pHlourin in *S. mutans* cannot only be used to analyze acid production by *S. mutans*, but also demonstrated a promising application in monitoring local microenvironment pH changes within multi-species biofilms. The latter might reflect the metabolic interactions between *S. mutans* and its neighboring bacterial species within oral communities.

The data presented in this paper demonstrated that the ecliptic pHluorin is a valuable tool for *in vivo/in situ* monitoring of acid production by the cariogenic *S. mutans* in a multispecies environment. The surface-anchored ecliptic pHluorin in *S. mutans* has a number of unique properties: (1) it is genetically encoded and biosynthesized *in situ*; (2) it is localized onto the cell surface of *S. mutans* and allows fluorescence imaging of microenvironment pH with high spatial and temporal resolution *in vivo*; (3) in combination with fluorescently labeled anti-*S. mutans* monoclonal antibody, we can effectively study the spatial distribution of *S. mutans* in correlation with its pH profile without worrying about the diffusion and penetration problem encountered when using the pH sensitive dyes; and (4) it does not interfere with normal structure and physiological processes of biofilm. In addition to its value for basic research in the acid-associated pathogenic processes involved in oral caries disease, the *S. mutans* strain could also be practical tool for evaluating the efficacy of dental products in eliminating *S. mutans* or reducing its acidogenicity.

In summary, we have developed a novel technique for optical imaging of pH in oral biofilms. Using this tool, we were able to demonstrate the faster pH drop observed within microcolonies than that of the surrounding medium following sucrose fermentation. Furthermore, we revealed the heterogeneity of pH profiles within *S. mutans* biofilms which reflected the differences in microbial metabolic activity. These results are consistent with similar findings by other group [12]. Our studies strongly support the notion that the ecliptic pHluorin is a valuable tool for *in vivo/in situ* monitoring of acid-associated pathogenicity of *S. mutans*.

## Supporting Information

Figure S1 **Schematic drawing of construction of pFW5-** ***ldh_p_*** **-leading sequence -** ***gfp*** **-linker-** ***spaP*** **.**
(DOC)
