# Supplementary material for: Investigating Acid Production by Streptococcus mutans with a Surface-Displayed pH-Sensitive Green Fluorescent Protein
Source: PLoS One. 2013 Feb 28;8(2):e57182. doi: 10.1371/journal.pone.0057182 (PMC3585301; doi:10.1371/journal.pone.0057182)

1. PCR amplification of four fragments


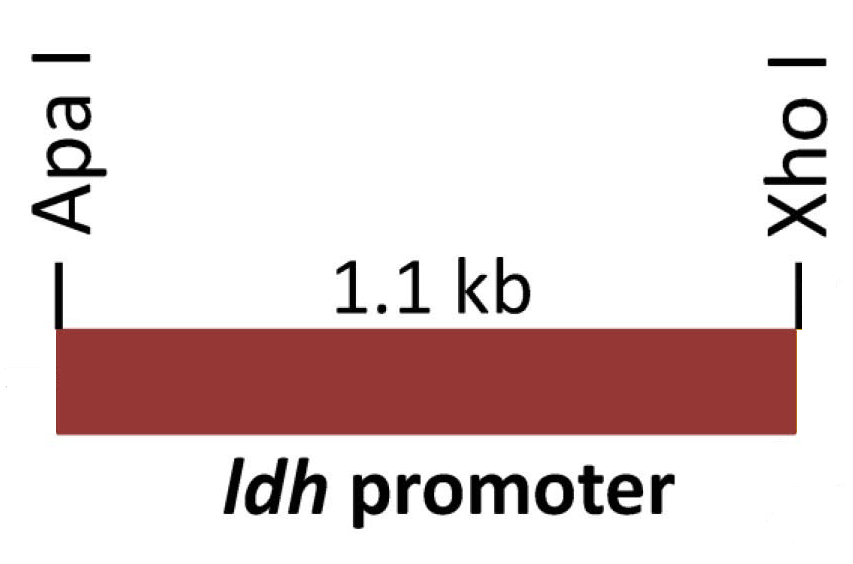

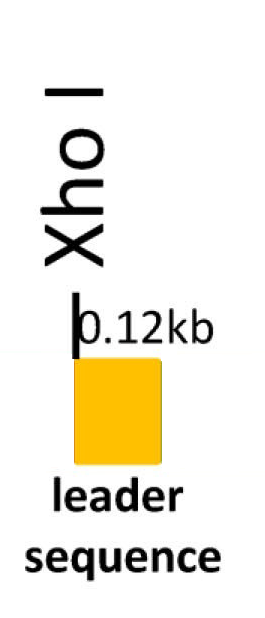

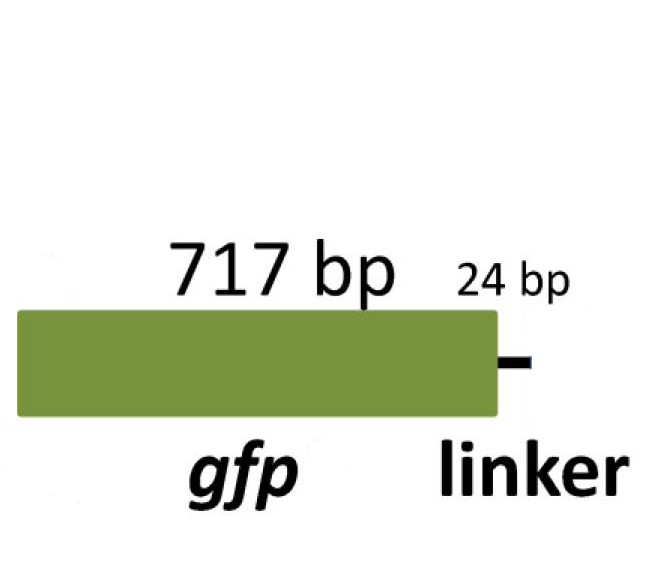

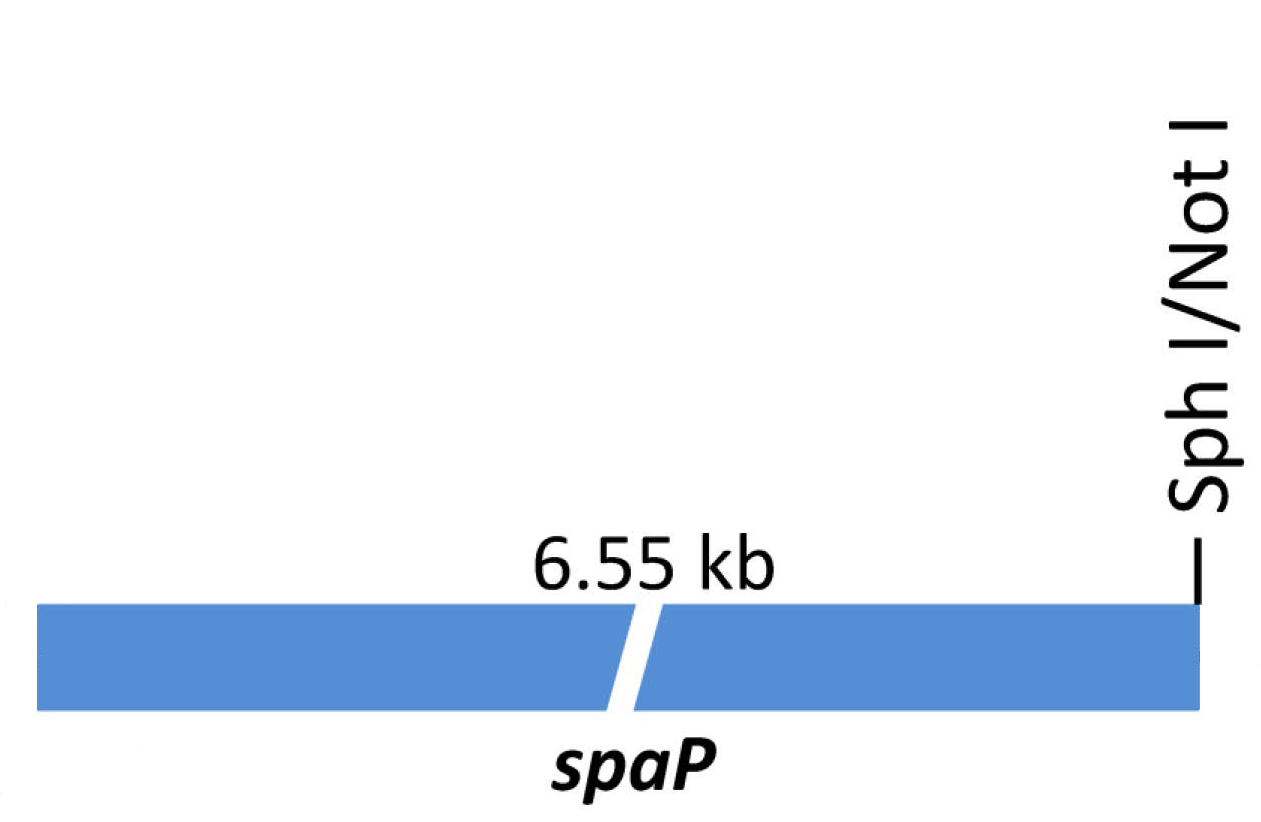


1. Overlap PCR


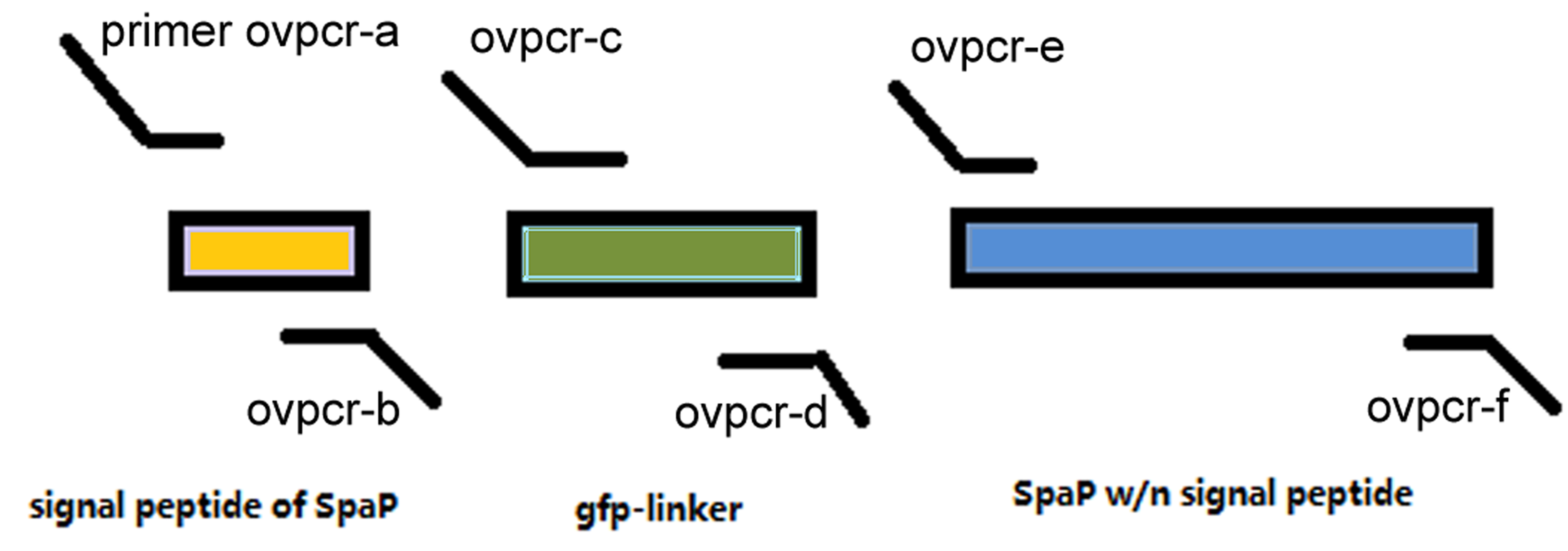


(3) Construction of pFW5-*ldh_p_*-leading sequence -*gfp*-linker-*spaP*


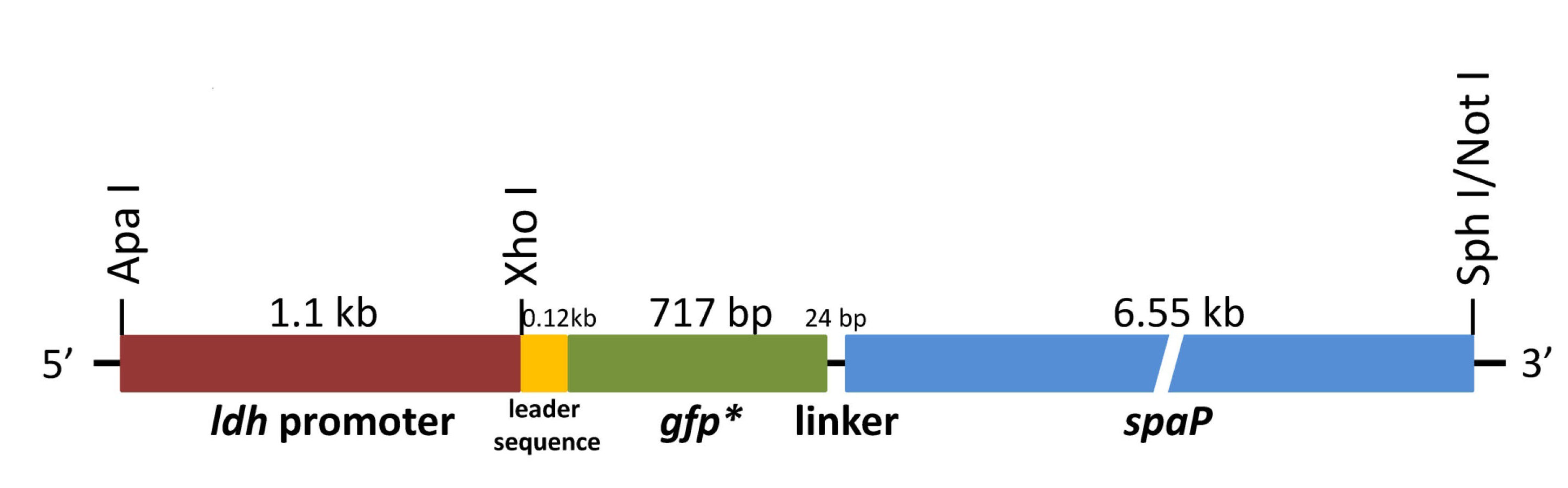

Supplement: Figure S1 — Schematic drawing of construction of pFW5- ldhp -leading sequence - gfp -linker- spaP . (DOC) [file pone.0057182.s001.doc]
